# Supplementary material for: Boosting Electrocatalytic Oxidation of Formic Acid on Ir(IV)-Doped PdAg Alloy Nanodendrites with Sub-5 nm Branches
Source: Molecules. 2023 Apr 23;28(9):3670. doi: 10.3390/molecules28093670 (PMC10180118; doi:10.3390/molecules28093670)
Supplement: Supplementary file 1 [file molecules-28-03670-s001.zip › molecules-2315480-supplementary.pdf]

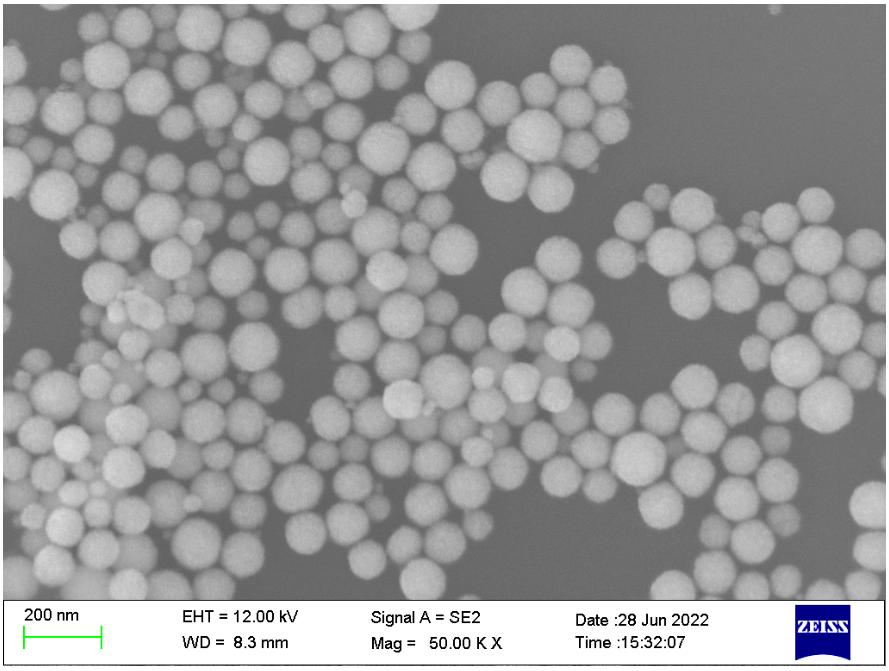

Figure S1. SEM image of PdAg NDs.

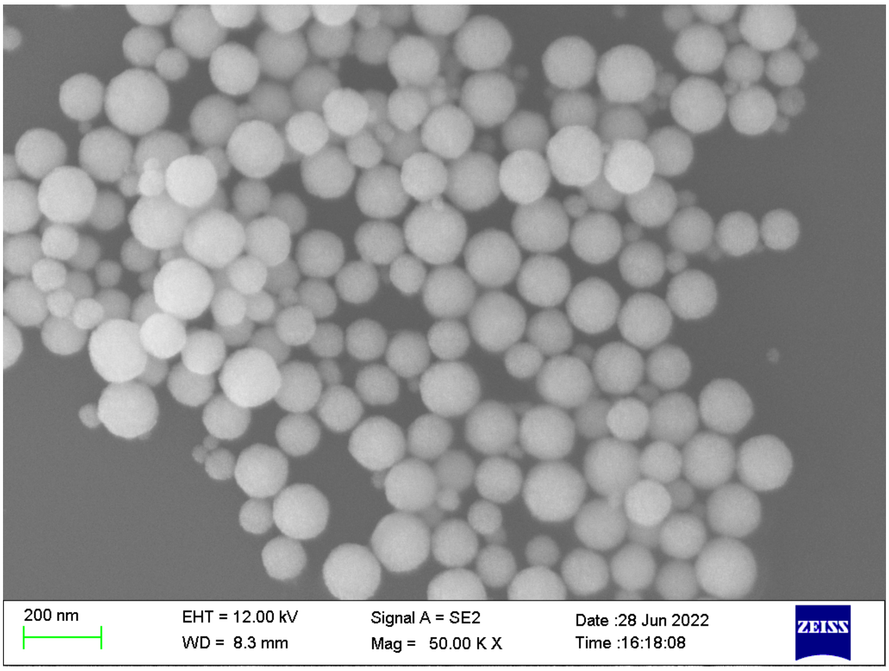

Figure S2. SEM image of Ir(IV)-doped PdAg NDs.

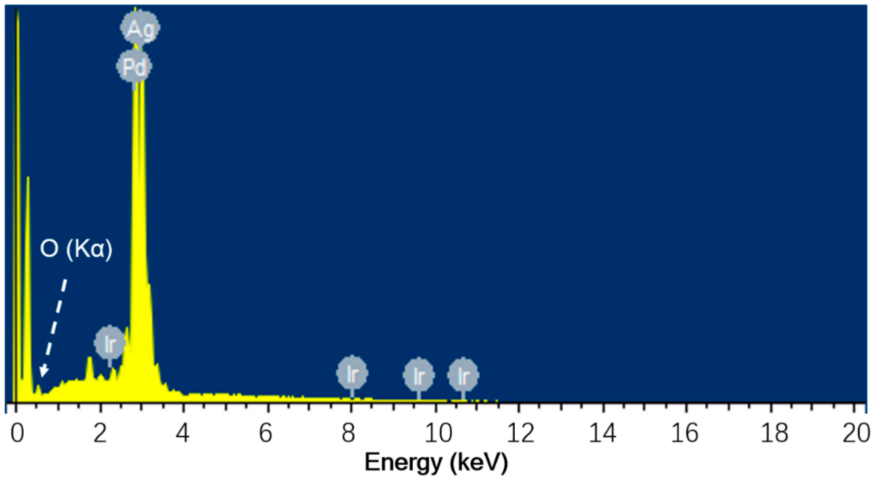

Figure S3. EDS spectrum of Ir(IV)-doped PdAg NDs.

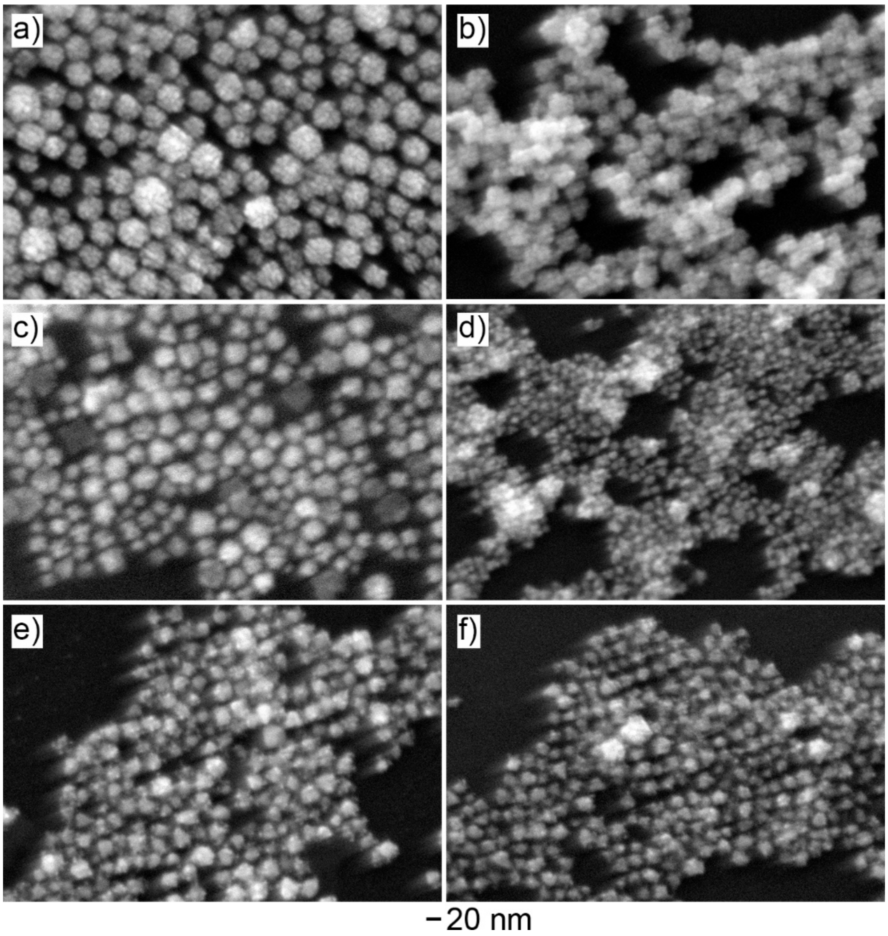

Figure S4. SEM images of Ir(IV)-doped PdAg NDs obtained *via* the standard procedure, except that the capping agent OTAC was replaced by a) CTAC, b) DCTAC, c) DDTAC, d) BC, e) CTAB, and f) OTAB, respectively, at the same concentrations.

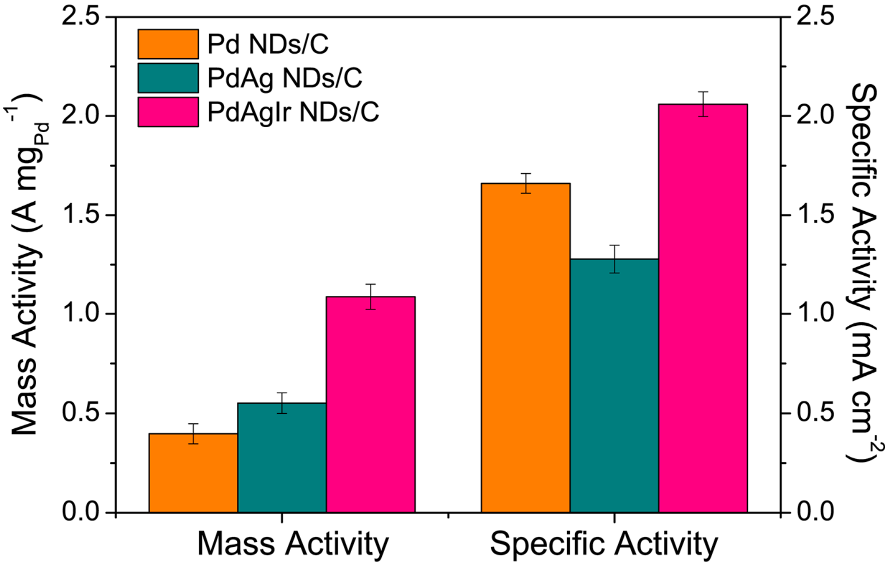

Figure S5. Histograms comparing the mass activity and specific activity of Pd-based electrocatalysts.

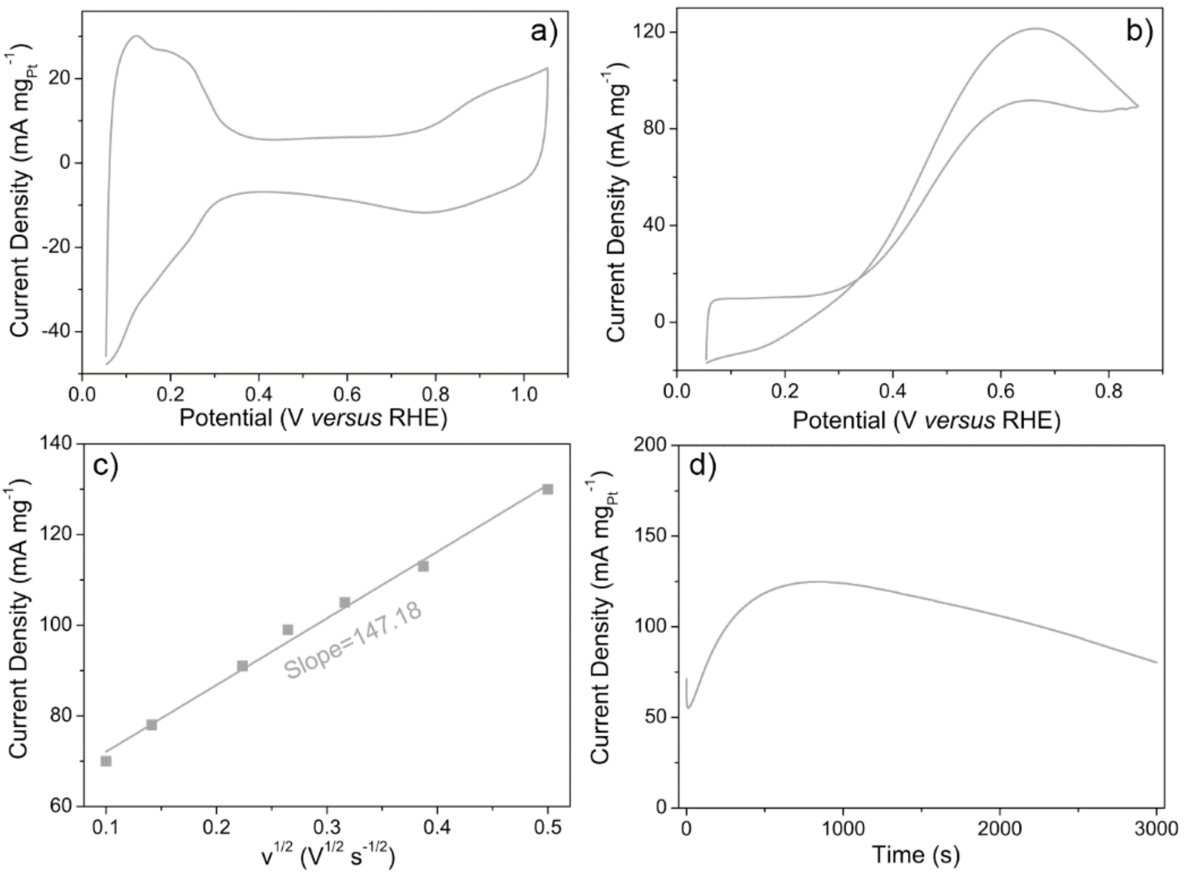

Figure S6. FAOR measurements of Pt/C: a) CV curves in N_2-saturated 0.1 M $HClO_4$ solution; b, c) CV curves in N_2-saturated 0.5 M FA+0.1 M $HClO_4$ solution; c) plots showing FAOR kinetics *versus* scan rates; d) *i-t* curves measured at 0.65 V vs. RHE.

Table S1. Summary of possible elementary steps and pathways in formic oxidation reaction.

No.	Elementary Step	Pathway
1	$HCOOH(g) + {}^{*} \rightarrow HCOO^{*} + H^{+} + e^{-}$	direct oxidation of FA *via* formate
2	$HCOO^{*} \rightarrow CO_2(g) + {}^{*} + H^{+} + e^{-}$	
3	$HCOOH(g) + {}^{*} \rightarrow COOH^{*} + H^{+} + e^{-}$	direct oxidation of FA *via* carboxyl
4	$COOH^{*} \rightarrow CO_2(g) + {}^{*} + H^{+} + e^{-}$	
5	$HCOOH(g) + {}^{*} \rightarrow COOH^{*} + H^{+} + e^{-}$	indirect oxidation of FA *via* carboxyl
6	$COOH^{*} + {}^{*} \rightarrow CO^{*} + OH^{*}$	
7	$CO^{*} + OH^{*} \rightarrow CO_2(g) + H^{+} + e^{-}$	
8	$COOH^{*} + H^{+} + e^{-} \rightarrow CO^{*} + H_2O(g)$	
9	$H_2O(g) + {}^{*} \rightarrow OH^{*} + H^{+} + e^{-}$	

Table S2. XRD results and theoretical diffraction peak positions of the PdAg NDs before and after the GRR treatment.

		Diffraction Peak Position (°)			
		(111)	(200)	(220)	(311)
PdAg NDs before GRR n(Pd):n(Ag)=58.5:41.5	XRD	39.0	45.6	66.4	80.5
	Theoretical*	39.3	45.7	66.6	80.2
PdAg NDs after GRR n(Pd):n(Ag):n(Ir)= 54.4:35.9:9.7	XRD	39.1	45.8	66.5	80.2
	Theoretical*	39.4	45.9	66.9	80.6
Pd	JCPDS No. 46-1043	40.1	46.7	68.1	82.1
Ir	JCPDS No. 06-0598	40.7	47.3	69.1	83.4
Ag	JCPDS No. 04-0783	38.1	44.3	64.4	77.5

*Theoretical values are calculated according to Vegard's Law.

Table S3. Summary of XRD results, crystalline size, and particle size of samples displayed in current work.

Sample	Two Theta (°)	Full width at half maxima (FWHM, °)	Crystalline Size* (nm)	Particle Size* (nm)
PdAg NDs before GRR	39.0	0.566	14.8	3.9 (branch)
PdAg NDs after GRR	39.1	0.607	13.8	4.3 (branch)

*The crystalline size was calculated using Debye-Scherrer equation and the particle size was statistically measured by counting 100 particles in corresponding typical SEM images. The difference between them should be attributed to the anisotropic morphology of current products and the presence of internal hierarchical structure.

Table S4. Summary of the relative peak areas (%) for each split B.E. peak and the parameters used to fit the Pd 3d, Ag 3d, and Ir 4f high-resolution XPS spectra.

Sample	Orbital	B. E. Peak (eV)	FWHM (eV)	peak area (a. u.)	element/oxidation state
PdAg NDs before GRR	Pd 3d	339.8	1.12	13092.3	Pd(0)
		341.8	5.97	6816.4	Pd(II)
		334.4	0.96	9340.3	Pd(0)
		335.3	2.27	7402.8	Pd(II)
	Ag 3d	373.1	0.82	12210.3	Ag(0)
		368.1	1.48	2118.2	Ag(I)
		367.1	0.78	17131.6	Ag(0)
		374.2	1.99	1677.5	Ag(I)
PdAg NDs after GRR	Pd 3d	340.1	1.09	12223.8	Pd(0)
		342.2	5.84	6030.4	Pd(II)
		334.7	0.94	8807.9	Pd(0)
		335.6	2.27	6501.8	Pd(II)
	Ag 3d	373.4	0.86	12501.7	Ag(0)
		368.5	1.56	1881.0	Ag(I)
		367.4	0.80	17021.0	Ag(0)
		375.4	3.04	1650.3	Ag(I)
	Ir 4f	65.2	/	/	Ir(IV)
		62.3	/	/	Ir(IV)

Table S5. Summary of FAOR performance for PdAg-based electrocatalysts.

Electrocatalyst	onset potential (mV *vs*. RHE)	peak potential (mV *vs*. RHE)	ECSA (m^2 g_{Pd}^{-1})	j_r/j_f	mass activity (mA mg^{-1})	specific activity (A m^{-2})	*j*(t=3000s) (mA mg^{-1})	*j*(t=3000s) (A m^{-2})
Pd NDs/C	75	511	23.90	0.85	396.9	16.6	96.5	4.0
PdAg NDs/C	91	662	43.16	0.91	551.4	12.8	22.7	0.53
PdAgIr NDs/C	74	736	52.72	0.95	1086	20.6	320.4	6.3

Table S6. Comparison in FAOR electrocatalytic performance for typical Pd-based electrocatalysts documented in literature.

**No.**	**Elemental Composition**	**Structure/ Morphology**	**Electrolyte**	**Scan Rate** (mV s^{-1})	**Mass Activity (mA mg_{Pd}^{-1})**	**Ref.**
1	PdCu	Curved Porous Metallene	0.5 M H_2SO_4 + 0.5 M FA	50	905	(ACS Appl. Mater. Interfaces, 2023) [1]
2	PdCu	Ordered Intermetallic Nanoparticles	0.5 M H_2SO_4 + 0.5 M FA	50	665	(Chem. Mater., 2022)[2]
3	PdCu	Coral-like alloy nanoparticles	0.5 M H_2SO_4 + 0.5 M FA	50	1050	(ACS Sustain. Chem. Eng., 2019)[3]
5	Pd_3Au	Quasispheroidal Nanoparticles	0.1 M $HClO_4$ + 0.1 M FA	20	520.6	(ACS Catal., 2014)[4]
6	PdRu	nanospine assemblies	0.5 M H_2SO_4 + 0.5 M FA	50	1100	(J. Mater. Chem. A, 2018)[5]
7	Ir(IV)-doped PdAg	nanodendrites	0.1 M $HClO_4$ + 0.1 M FA	50	1086	current work

Table S7. Information of chemicals used in current study.

Name	Formula/Model	Purity	Supplier	Location
sodium tetrachloropalladate(II)	Na_2PdCl_4	99.9%	Aladdin Chemical	Shanghai China
silver nitrate	$AgNO_3$	⩾99.0%		
iridium(III) chloride	$IrCl_3$	99.8% metals basis		
ascorbic acid	AA	99.0%		
hexadecyltrimethylammonium chloride	CTAC	97%		
octadecyltrimethylammonium bromide	OTAB	98%		
octadecyltrimethylammonium chloride	OTAC	90%	Lusen Chemical	Linyi China
dodecyltrimethylammonium chloride	DDTAC	98%		
docosyltrimethylammonium chloride	DCTAC	85%	Degussa AG	Germany
carbon black	XC-72	/	Cabot Corp.	USA
formic acid	HCOOH	⩾98.0%	Sinopharm	Shanghai China
perchloric acid	$HClO_4$	70.0～72.0%		
Nafion	/	5% wt%, ethanol solution	Cool Chemistry	Beijing China

References

[1] L. Zhang, Z. Zhao, X. Fu, S. Zhu, Y. Min, Q. Xu, Q. Li, Curved Porous PdCu Metallene as a High-Efficiency Bifunctional Electrocatalyst for Oxygen Reduction and Formic Acid Oxidation, ACS Appl. Mater. Interfaces, 2023, 15, 5198-5208.

[2] X. Li, Y. Liu, J.-J. Zhang, B. Yan, C. Jin, J. Dou, M. Li, X. Feng, G. Liu, No Annealing Synthesis of Ordered Intermetallic PdCu Nanocatalysts for Boosting Formic Acid Oxidation, Chem. Mater., 2022, 34, 1385-1391.

[3] J. Zheng, H. Zeng, C. Tan, T. Zhang, B. Zhao, W. Guo, H. Wang, Y. Sun, L. Jiang, Coral-like PdCu Alloy Nanoparticles Act as Stable Electrocatalysts for Highly Efficient Formic Acid Oxidation, ACS Sustan. Chem. Eng., 2019, 7, 15354-15360.

[4] S.-Y. Lee, N. Jung, J. Cho, H.-Y. Park, J. Ryu, I. Jang, H.-J. Kim, E. Cho, Y.-H. Park, H.C. Ham, J.H. Jang, S.J. Yoo, Surface-Rearranged Pd_3Au/C Nanocatalysts by Using CO-Induced Segregation for Formic Acid Oxidation Reactions, ACS Catal., 2014, 4, 2402-2408.

[5] H. Wang, Y. Li, C. Li, Z. Wang, Y. Xu, X. Li, H. Xue, L. Wang, Hyperbranched PdRu nanospine assemblies: an efficient electrocatalyst for formic acid oxidation, J. Mater. Chem. A, 2018, 6, 17514-17518.
